# Reproductive Health Literacy and Fertility Awareness Among Polish Female Students

**DOI:** 10.3389/fpubh.2020.00499

**Published:** 2020-09-11

**Authors:** Ewelina Chawłowska, Agnieszka Lipiak, Jana Krzysztoszek, Beata Krupa, Rafał Staszewski

**Affiliations:** ^1^Laboratory of International Health, Department of Preventive Medicine, Poznan University of Medical Sciences, Poznań, Poland; ^2^Department of Physical Activity Teaching, Poznan University of Physical Education, Poznań, Poland; ^3^Independent Researcher, Poznan, Poland; ^4^Department of Hypertensiology, Angiology and Internal Diseases, Poznan University of Medical Sciences, Poznań, Poland

**Keywords:** knowledge, health literacy, fertility awareness, reproductive health, Poland, young women, students

## Abstract

The falling fertility indicators and high prevalence of infertility in Poland make it important for people of reproductive age to have good knowledge of their own fertility in order to be able to take care of their reproductive health. This paper examines reproductive health literacy and fertility awareness among Polish female students. It can help identify gaps in reproductive health education in Poland. The study group included 456 women aged 18–29, who were students of 6 public universities located in Poznan, Poland. The method used was a survey using a self-developed questionnaire assessing the students' knowledge of female and male fertility-related physiology and fertility patterns. The respondents' knowledge was assessed on the basis of the percentage of correct answers. Regression analysis and univariate analysis of variance were used to explore relationships between the students' knowledge and their age, year of study, university and source(s) of information. The average score of correct answers was 55.8%. Older students and medical university students were the most knowledgeable. 93.4% of the respondents correctly identified the optimum age for a woman to have the first child from the point of view of achieving pregnancy fast. Over 90% of the respondents knew such fertility-compromising risks as smoking, diseases and psychological distress. There was much poorer awareness of the adverse effect of unbalanced diet, irregular sleep, and long-lasting physical effort. 47.1% of the students reported gaining information from a number of sources, but as many as 28.3% said their only source was primary or middle school classes. Reproductive health knowledge among the young female students is incomplete, especially as regards lifestyle-related risks. They should be encouraged to supplement it by consulting reliable sources such as health professionals. It is advisable to ensure that the curricula of medical university students provide thorough knowledge in this area, and to arrange suitable electives for students from other universities. As primary and secondary school classes remain an important source of information, quality teaching at these levels should be offered with a focus on making the knowledge as practical and operational as possible. Relevant graduate, postgraduate and in-service courses should be available to professionals responsible for spreading reproductive health knowledge.

## Introduction

Fertility and reproductive health (RH) are important aspects of life, both for people of reproductive age and for the whole community. In Europe, the total fertility rate (TFR) has been falling within the last couple of decades, contributing to the new demography of Europe—a rapid ageing of the region (1). The trends for Poland are no different. The TFR for Poland has been below 1.5 since 1997 (2) and is expected to remain at the sub-replacement level (below 2.1). As a result, the age structure of the population is changing, leading to a steady growth of the economic old age dependency ratio, i.e., the ratio between the inactive elderly aged 65+ and the number of the employed. It is projected to rise in the whole EU from 43.1% in 2016 to 68.5% in 2070, but Poland is to reach the highest rate of all Member States (92.5%) (1).

One of the reasons behind such trends is delayed childbearing, which may be a risk factor for adverse pregnancy outcomes and pregnancy complications (3–5). Although advanced maternal age is associated with a number of health-related and developmental benefits (6, 7), it also contributes to higher prevalence of infertility, growing need for infertility treatment and assisted reproductive technology (ART), involuntary childlessness, and the resulting serious psychological distress of infertile couples (8–11). There are no current data available on the prevalence of infertility in Poland. It is estimated to be similar to the prevalence observed in other developed countries and affect 15–20% of all couples (12, 13). However, there are studies showing the scale of involuntary childlessness and the main reasons behind it. The mean personal ideal number of children for Poles aged 25–39 years is 2.12 (women) and 1.99 (men), while the actual numbers are 1.27 and 0.82, respectively, which demonstrates a considerable fertility gap between ideals and life (14). A vast majority of people of reproductive age in Poland have childbearing intentions; only 13% of childless men and 12% of childless women aged 18–39 interviewed in 2014 intended to remain childless (15). In the group of childless people who intended to have children within the next 3 years, only 33% of men and 34% of women succeeded, 39% of women and 40% of men postponed parenthood, and 26% of women and 27% of men abandoned their plans (16). The most important barriers to having the first child faced by Poles aged 20–39 years turned out to be the lack of partner (27.8%), low standard of living (22.8%), infertility (14.4%), and uncertain future (8.8%). The top barriers to having the second child are low standard of living (31.8%), infertility (12.2%), uncertain future (11.1%), and high costs of raising children (7.9%). The importance of economic barriers grows with an increasing number of children, while the importance of infertility grows with increasing age and, strikingly, with decreasing education level (17, 18). Young Poles of both sexes are more willing to become parents when they have stable and regular income. Young Polish mothers are more willing to become mothers again when they feel they are able to reconcile family and work life and are supported by their partners in everyday chores (19). In a study of childless Polish women aged 37–46 years, 56% of the respondents had no stable partner, but among those who had partners and wanted to have children, the most important reasons for remaining childless were problems getting pregnant (23.7%) or other health problems such as chronic illnesses or disabilities (21.2%) (20). Thus, the three recurrent modifiable factors affecting childbearing in Poland seem to be (i) economic instability, (ii) work-family tensions, and (iii) health problems (including infertility). Given the above, there is a need for comprehensive social and public health policies that could reduce involuntary childlessness and the related distress at an individual level, and at the same time alleviate population ageing at the societal level. The policies cannot address such issues as the lack of an appropriate partner, but can and should aim to, (i) support economically stable work and living settings, (ii) promote gender equality and work-family reconciliation, and last but not least, (ii) intensify health education and promotion, particularly with respect to RH.

Taking care of one's RH pertains to a wide range of areas, such as general care for one's health, obtaining detailed information on RH physiology, increasing one's fertility awareness (FA), i.e., learning to identify fertile and infertile phases of a woman's menstrual cycle, as well as avoiding factors with adverse impact on RH. Having sound knowledge in this domain is crucial for making informed decisions and shaping healthy attitudes and practices.

Young female students are the one demographic group for which the knowledge in the field of human fertility is essential, for two important reasons. Firstly, many of them are going to have children in the near future, which is why it is important for them to know how their reproductive system works. Secondly, they will soon graduate, which means that they are about to be among the best educated young people in Poland. It is, by the way, quite a populous group, as 53.7% of Polish women aged 25–34 (21) and 52% of the women who gave birth in 2016 (22) have tertiary education. Therefore, their competence should not be limited to the area of their studies, but should extend to other areas, in particular to those directly related to their own health and well-being of the families they are going to build. Considering all the aspects discussed above, we believed it would be interesting to explore RH knowledge among Polish female university students.

## Materials and Methods

### Participants

The study included a group of 456 women aged 18–29 (mean age = 21.95 ± 2.45 years), who were students of higher educational institutions and came from rural (26.87%) and urban (73.13%) areas throughout Poland. 98.9% of the participants were nulliparous, whereas 1.1% had children. Only 1 of the 5 parous participants declared that her pregnancy had been intended. The survey was conducted in Poznan, one of the largest university cities in Poland, at 6 public universities: Poznan University of Medical Sciences (*n* = 178), Poznan University of Life Sciences (*n* = 58), Poznan University of Economics and Business (*n* = 58), Academy of Music in Poznan (*n* = 31), Poznan University of Technology (*n* = 55), and Adam Mickiewicz University (*n* = 76). The criteria for selecting women to participate in the survey were: (i) age between 18 a 29 years; (ii) being a current student. Prior to the study, each respondent had been informed of the purpose of the study, the entity responsible for carrying it out, the way the results would be used, as well as the voluntary and anonymous nature of participation.

### Research Tool

The research method used was a survey. The respondents were interviewed face to face with the use of a self-developed questionnaire composed of 20 questions: 18 closed-ended ones (2 yes/no questions, 14 disjunctive multiple choice questions, and 2 conjunctive multiple choice questions), 1 semi-open question and 1 open question. Seventeen of the twenty questions assessed the respondents' knowledge of female and male fertility-related physiology and fertility patterns. Two questions determined the respondents' maternity status. One question explored the source(s) of the respondents' fertility knowledge. There was also a separate part with questions establishing the respondents' demographic and social details. An English version of the questionnaire is attached as Additional File 1.

### Data Analyses

The respondents' knowledge was assessed on the basis of the percentage of correct answers to individual questions. Where not indicated otherwise, the percentages given below are the proportions of correct answers in the whole study group. Whenever a respondent failed to provide an answer, it was regarded as an incorrect answer. After the initial computational analyses of the socio-demographic characteristics of the study sample as well as calculations of the proportions of correct answers in particular subject areas, further analyses were carried out with use of STATISTICA *Project file Version 10*. Univariate analysis of variance (ANOVA test) was performed to explore possible relationships between the students' knowledge and their age, university and source(s) of information. *P*-values of *p* ≤ 0.05 were considered significant. Multiple regression analysis was used to estimate the effect of age and year of study on the respondents' knowledge.

## Results

**Figure 1:**
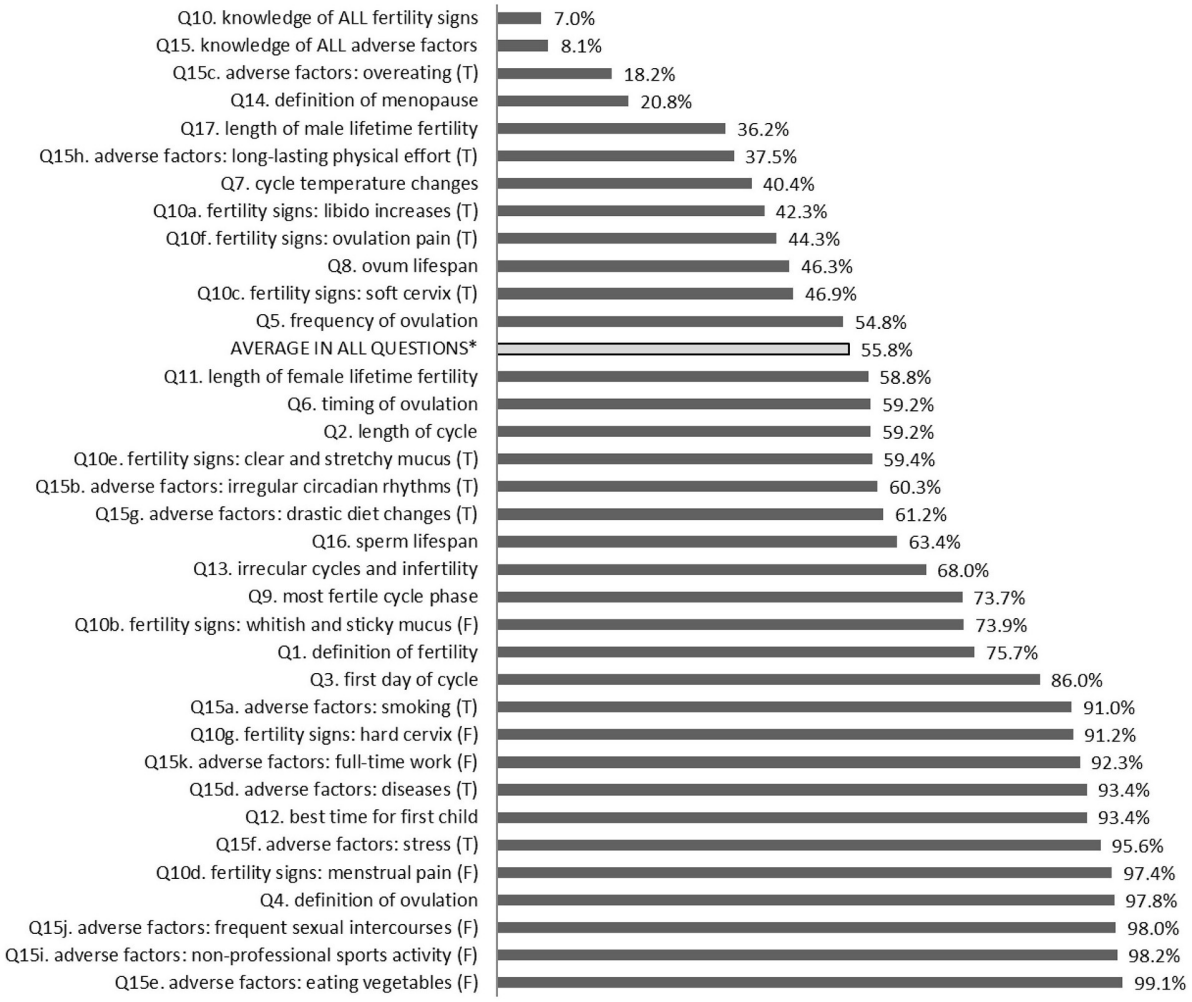
Percentages of correct answers to particular questionnaire questions. *Excluding the results in the subquestions 10a-g and 15a-k; *T, true; F, false*.

55.8% of the answers to the 17 knowledge-related questions were correct (see Figure 1). The average individual score was 9.49 points out of 17 (55.8%), and the median individual score was 9. As regards the knowledge of different age groups within the study group, the percentages were as follows: 1st group (18–21 years old) – 52.9%, 2nd group – (22–24 years old) – 57.6%, and 3rd group (25–29 years old) – 60.3%. To estimate how age and year of study influenced the respondents' knowledge, multiple regression analysis was used. The model turned out significant [*F*_(2,449)_ = 13.565; *p* < 0.0001], and the two predictors together accounted for only 6% of the variance in knowledge (*R*^2^ = 0.057). The influence of the year of study was found statistically significant (β = 0.23; *t* = 3.128, *p* < 0.0001), but the influence of age was not (β = 0.01; *t* = 0.117, *p* > 0.05).

None of the respondents replied correctly to all the questions. It should be noted that the questionnaire was not an easy one. The two conjunctive multiple choice questions, each having a set of correct answers, were particularly demanding: the question regarding symptoms of ovulation, and the question about fertility-affecting factors. Only fully correct answers were counted as correct, also in respect of the two questions. If the hardest questions were not taken into account, the proportion of correct answers (and the mean score) would reach 62.2%. Rather than rate these results as satisfactory or unsatisfactory, the authors would like to point to the areas which turned out particularly difficult to the participants, as well as discuss the questions presenting statistically significant differences in knowledge between age groups, universities and sources of information.

As it has already been mentioned above, the questions which presented a big challenge to the participants were those which required indicating a whole set of correct answers. The first of them, question no. 10 (Q10), referred to fertility signs, i.e., a set of symptoms characteristic for the most fertile period of the cycle. There were seven possible symptoms (Q10a–Q10g) to choose from conjunctively, including 4 correct ones. Only 7.0% (*n* = 32) of the respondents managed to identify the whole set correctly, the vast majority of them (*n* = 27) attending the medical university. However, if the questions about the symptoms were treated as seven separate subquestions, the share of correct answers for specific symptoms would range from 42.3–97.4%, and the mean for all the symptoms would be 65.1% (see Table 1).

**Table 1:** Knowledge of fertility signs in different age groups.

Knowledge^*^ of:	**Age (percentage of participants in a given age group)**						
	**Mean**	**18–21**	**22–24**	**25–29**			
	***n* = 456**	**(42.8%)**	**(48.5%)**	**(8.8%)**	***p***	***SD***	***SE***
Q10a. fertility signs: libido increases (T)	42.3%	38.50%	43.40%	55.00%	> 0.05	0.49	0.02
Q10b. fertility signs: whitish and sticky mucus (F)	73.9%	66.20%	79.20%	82.50%	**≤0.005**	0.44	0.02
Q10c. fertility signs: soft cervix (T)	46.9%	41.50%	51.60%	47.50%	> 0.05	0.50	0.02
Q10d. fertility signs: menstrual pain (F)	97.4%	95.90%	98.60%	97.50%	> 0.05	0.16	0.01
Q10e. fertility signs: clear and stretchy mucus (T)	59.4%	51.30%	65.20%	67.50%	**≤0.05**	0.49	0.02
Q10f. fertility signs: ovulation pain (T)	44.3%	40.50%	46.60%	50.00%	> 0.05	0.50	0.02
Q10g. fertility signs: hard cervix (F)	91.2%	87.70%	93.20%	97.50%	> 0.05	0.28	0.01

* *As a proportion of correct answers*.

*T, true; F, false*.

*p ≤ 0.05 (in bold) were considered significant*.

Statistically significant differences were found between the 3 age groups as regards the knowledge of fertile cervical mucus characteristics: the older the students were, the more often they gave correct answers. The respondents were better at identifying the symptoms that are *not* present during the fertile phase (the cervix is *not* hard, cervical mucus is *not* whitish or sticky, menstrual pain does *not* occur) than at indicating those that can actually be observed (libido increases, the cervix is soft, cervical mucus is clear and stretchy, ovulation pain occurs). Most of the students did not know that libido was heightened during the most fertile phase.

The second question that proved problematic was the one about the factors adversely affecting human reproductive potential (Q15). The list included 11 factors (Q15a–Q15k), 7 of which were correct. All the factors were identified correctly by only 8.1% (*n* = 37) of the interviewees, most of them from the University of Medical Sciences and the University of Life Sciences. Again, with this question treated as 11 separate subquestions, the scores for specific factors range from 18.2 to 99.1%, and the average score for all the factors reaches 76.8% (see Table 2).

**Table 2:** Knowledge of the factors which may adversely affect fertility in different age groups.

Knowledge^*^ of:	**Age (percentage of participants in a given age group)**						
	**Mean**	**18–21**	**22–24**	**25–29**			
	***n* = 456**	**(42.8%)**	**(48.5%)**	**(8.8%)**	***p***	***SD***	***SE***
Q15a. adverse factors: smoking (T)	91.0%	91.8%	90.5%	90.0%	> 0.05	0.29	0.01
Q15b. adverse factors: irregular circadian rhythms (T)	60.3%	46.7%	69.2%	75.0%	**≤0.001**	0.49	0.02
Q15c. adverse factors: overeating (T)	18.2%	11.3%	22.6%	25.0%	**≤0.05**	0.39	0.02
Q15d. adverse factors: diseases (T)	93.4%	92.8%	93.7%	95.0%	>0.05	0.25	0.01
Q15e. adverse factors: eating vegetables (F)	99.1%	100.0%	98.6%	97.5%	> 0.05	0.09	0.00
Q15f. adverse factors: stress (T)	95.6%	93.3%	96.8%	100.0%	> 0.05	0.21	0.01
Q15g. adverse factors: drastic diet changes (T)	61.2%	55.9%	64.3%	65.0%	> 0.05	0.49	0.02
Q15h. adverse factors: long-lasting physical effort (T)	37.5%	28.7%	42.5%	52.5%	**≤0.001**	0.48	0.02
Q15i. adverse factors: non-professional sports activity (F)	98.2%	100.0%	97.3%	92.5%	**≤0.005**	0.13	0.01
Q15j. adverse factors: frequent sexual intercourses (F)	98.0%	99.0%	97.3%	97.5%	> 0.05	0.14	0.01
Q15k. adverse factors: full-time work (F)	92.3%	92.8%	92.3%	92.5%	> 0.05	0.27	0.01

* *As a proportion of correct answers*.

*T, true; F, false*.

*p ≤ 0.05 (in bold) were considered significant*.

The students had excellent knowledge of a few risks (stress, diseases, smoking), but much poorer knowledge of other factors (overeating, irregular circadian rhythms, long-lasting physical effort). The awareness of the latter factors was directly proportional to the age of the respondents. Interestingly, in the question about non-professional sports activity the proportions were inverted, i.e., the younger the respondents were, the more correct they were in claiming that such activity does not have an adverse effect on fertility. The analysis of the sources of information showed that the women seeking information from professional sources had slightly more accurate knowledge of the fertility-compromising factors (see Additional File 2).

The next difficult question was the one where the students were asked to choose the right definition of menopause (Q14). The average percentage of correct answers was only 20.8%. The respondents' age influenced their awareness—the older they were, the more they knew about it. Detailed differences between the age groups with respect to this question, as well as to other questions where the differences were statistically significant, are presented in Table 3.

**Table 3:** Knowledge differences between age groups.

	**Age (percentage of participants in a given age group)**						
	**Mean**	**18–21**	**22–24**	**25–29**			
Knowledge^*^ of:	***n* = 456**	**(42.8%)**	**(48.5%)**	**(8.8%)**	p^**^	***SD***	***SE***
Q1. definition of fertility	75.7%	72.8%	80.1%	65.0%	≤ 0.05	0.62	0.03
Q3. first day of cycle	86.0%	83.6%	89.6%	82.6%	0.0408	0.61	0.03
Q10b. fertility signs: whitish and sticky mucus (F)	73.9%	66.2%	79.2%	82.5%	≤ 0.005	0.44	0.02
Q10e. fertility signs: clear and stretchy mucus (T)	59.4%	51.3%	65.2%	67.5%	≤ 0.05	0.49	0.02
Q14. definition of menopause	20.8%	12.8%	26.2%	30.0%	0.0037	1.06	0.05
Q15b. adverse factors: irregular circadian rhythms (T)	60.3%	46.7%	69.2%	75.0%	≤ 0.001	0.49	0.02
Q15c. adverse factors: overeating (T)	18.2%	11.3%	22.6%	25.0%	≤ 0.05	0.39	0.02
Q15h. adverse factors: long-lasting physical effort (T)	37.5%	28.7%	42.5%	52.5%	≤ 0.001	0.48	0.02
Q15i. adverse factors: non-professional sports activity (F)	98.2%	100.0%	97.3%	92.5%	≤ 0.005	0.13	0.01
AVERAGE IN ALL QUESTIONS^***^	55.8%	52.9%	57.6%	60.3%	–	0.15	0.01

* *As a proportion of correct answers*.

** *Only the statistically significant results are presented*.

*** *Excluding the results in the subquestions 10a-g and 15a-k*.

*T, true; F, false*.

Fifty-seven percent of the whole study group believed that menopause is the period in a life of a woman when her fertility gradually ceases. The medical students were the group with the highest percentage of correct answers (see Additional File 3). The poorest performance was recorded in the students who named parents as their source of information (no correct answers), whereas the best scores were observed in those who obtained information from health professionals and “other sources” (e.g., university courses, natural family planning courses, leaflets, siblings; see Additional File 2).

The question with a somewhat bigger proportion of correct answers (36.2%) was the one about the length of male fertility during a healthy man's life (Q17). By way of comparison, the percentage of correct answers to the question about the length of female fertility (Q11) was 58.8%. When asked about the lifespan of a sperm (Q16) and an ovum (Q8), the respondents had better knowledge on the male reproductive cell (63.4%) than on the female cell (46.3%).

A question concerning a more observable subject matter—the changes of basal body temperature (BBT) during the cycle (Q7)—yielded very diverse answers, depending on the source of information (see Additional File 2). While 40.4% on average gave correct answers, the proportion ranged from 87.5% in the subjects who relied on the information obtained from parents to 28.6% in those who gained it from peers.

When asked if ovulation occurs in every cycle (Q5), over a half of the respondents (54.8%) answered correctly. Medical students had much better knowledge (71.9%, see Additional File 3). The answers to the question about the timing of ovulation during the cycle (Q6) were very divergent across groups with different sources of information. The lowest percentage of correct responses (25.0%) was observed in the respondents informed by parents. They usually believed that ovulation occurs exactly in the middle of the cycle. The highest score (79.2%) was achieved by the women who based their answers on the information from the media (see Additional File 2). On average, 59.2% of all the answers to this question were correct.

The same proportion of correct answers (59.2%) was observed in the question about the length of a menstrual cycle (Q2). A quarter of the respondents were of the opinion that a cycle lasts 26–28 days, whereas the scope is a bit broader: 22–35 days. Given the fact that approximately 2 in 3 women have cycles which are 25–30 days long (23), the submitted answers may be based on the subjects' own experience.

When asked about the relation between irregular cycles and infertility (Q13), most of the respondents (68.0%) were aware that infertility is related to other factors apart from cycle length. The levels of knowledge in this area did not depend on any of the factors analysed in the present study.

73.7% of the respondents knew which cycle phase is the most fertile (Q9). Their knowledge varied depending on the university, with the medical university students scoring significantly better than others (82.0%, see Additional File 3).

In one question the respondents were asked to provide a definition for fertility (Q1). Only the answers fully conveying the meaning of the following definition, e.g., “the ability to reproduce,” were deemed correct. 75.7% of the interviewed females provided correct definitions, which seems quite a good result given the fact that it was an open-ended question. The 2nd age group had the highest number of correct answers, whereas the oldest group scored the lowest (see Table 3).

The majority of the study group knew on which day the female menstrual cycle begins (Q3), and the average share of correct answers reached 86.0%. It was the highest in the 2nd age group (89.6%, see Table 3), and among the medical school students (94.9%, see Additional File 3).

Also the vast majority of the respondents (93.4%) answered correctly when asked about the optimum age for a woman to give birth to the first child (Q12). All of the respondents who relied on the information from parents were correct about it, compared to only 71.4% of those informed by peers (see Additional File 2).

The question which turned out the easiest was the one where the respondents were asked to define ovulation (Q4)—the proportion of correct answers reached 97.8%. Once again, the medical students were the most knowledgeable (see Additional File 3). There were big differences between the groups using different sources of information. The proportion of correct answers equalled 100% in the women informed by health professionals and parents. The score was much lower (71.4%) in the respondents who identified peers as their source of information (see Additional File 2).

## Discussion

The general level of RH knowledge found in the present study is consistent with similar global research. In a systematic review that included 71 articles published worldwide between 1994 and 2017, Pedro et al. (24) compared the knowledge of people of reproductive age in the world and found the reported knowledge levels to be mostly low (<40% of correct answers) to average (40–59% correct answers). On this scale, the general knowledge of the respondents of the present study (55.8%) would be rated as average. Trying to identify the variables associated with different knowledge levels, the authors of the review reported generally higher levels in women, people of higher education, those having difficulty conceiving, and those who had planned their pregnancies. They were also higher among medical or health students than among students of other areas, which is consistent with the results of the present study (24).

As regards the detailed results of the present study, it seems that the knowledge of Polish female students is incomplete and patchy. Firstly, most of the respondents tend to have better knowledge in the areas either close to their own experience or relevant to them at a given time—perhaps the areas which they feel personally motivated to explore or which are likely to be discussed during patient—gynaecologist interactions. Since they are all in reproductive age, they are well-informed about the basic menstruation and ovulation facts such as which day is the first day of the menstrual cycle and which phase is the most fertile phase of the cycle. The findings are corroborated by a large study of 2019 conducted on 20,002 Polish women (mean age 27.7 years, 71% with higher education) (25), in which the questions about the first day of the menstrual cycle and the average length of the cycle had more than 90% correct answers. Similarly, in a study by Makara-Studzińska et al., 200 students of different Polish universities were well aware of the first day the female menstrual cycle (26). Also in a 2010 study by Deluga and Wiśniewska carried out among women aged 18–31 years, 90.3% of the interviewed females knew which day it was (27).

On the other hand, there are a few subjects where the study group had poor scores. Perhaps these were the areas remote from the participants' everyday experience or considered to be irrelevant for the time being, the areas where their personal motivation to seek information was weaker, and where their knowledge depended more on formal education. Thus, the questions with markedly better and markedly worse results identified in the present study may reflect, respectively, the areas of focus and neglect in RH education in Poland. For example, the participants had poor knowledge of menopause, which is a period still decades ahead for most of them. Similarly, their awareness of fertility signs was limited, though found to be generally increasing with age (see Table 1). Fertility signs were also a demanding subject for the participants of the study by Warzecha et al. (25)—they had the most difficulty answering the question about the time of the cycle when BBT increases (10.4% of correct answers). The young women presumably sought to avoid pregnancy rather than achieve it, and preferred such contraceptive methods (e.g., hormonal contraception) that made their own fertility signs absent or altered. According to Zgliczyńska et al., 51% of Polish female contraception users aged 18–35 years rely on hormonal contraception, while 13%—on natural family planning based on observing one's fertility signs (28). The present study was not concerned with practices, including contraceptive practices, so it is not possible to check if the students more knowledgeable about fertility signs used that knowledge for natural family planning. However, an American study of 2012 found that the respondents who used natural family planning or withdrawal as contraception had slightly better, though still inaccurate, awareness of fertility signs. These two groups seemed more interested in observing fertility symptoms (29).

Another topic where the respondents displayed fragmentary knowledge was the factors which may adversely affect fertility. Few of the participants were able to name them all. A number of factors (stress, diseases, and smoking) were identified correctly by the vast majority of the students. Surprisingly, some other risks (overeating, long-lasting physical effort, irregular sleep patterns) were selected markedly less often. It may seem that the young women do not realise how these lifestyle-related factors may influence their present and future lives. The fertility-compromising risk factors that were readily recognised may be among the behavioural risk factors often mentioned in other health-related contexts. The international review by Pedro et al. found good knowledge of lifestyle-related infertility risk factors (smoking, alcohol, and substance use) in most of the reviewed research and attributed it to the fact that they are common and generally well-recognised risk factors for other well-known chronic diseases such as cardiac disease and cancer. The awareness of these risk factors was generally higher in well-educated groups and in people trying to conceive (24). The members of the latter group were interviewed in a study carried out in 79 countries (83.2% women, 53.9% with university education), and the risk factor correctly identified by most of the participants was smoking, whereas the poorly recognised factors included sexually transmitted infections (STIs), age over 40 years and obesity in women, and mumps after puberty in men (30, 31). In a Canadian study among childless women aged 20–50 years (81% with at least college education), most participants were aware of the adverse effect of STIs (82.2%), and abnormal woman's weight (66.2%) (32). Fertility clinic patients interviewed by Homan and Norman readily identified such lifestyle-related risk factors as smoking, being over- or underweight, taking recreational drugs, and stress (33). By way of comparison, only 38% of the women not trying to conceive interviewed as part of the American Fertility IQ 2011 survey were aware that reproductive health may be affected by smoking and 21% knew the harmful effect of too much physical exercise, but a majority knew about the adverse effect of stress and abnormal weight (34).

Interestingly, the area where the participants of the present study scored relatively well was the knowledge of selected not directly observable fertility aspects. The vast majority knew that ovulation is “a release of an ovum from an ovarian follicle,” 63.4%—how long a sperm lives, and 46.3%—how long an ovum lives. The last question was also answered correctly by 44.3% of young women in another Polish study, with nearly 2/3 of the participants being university students (27). In the study by Warzecha et al., 62.5% of the young Polish women (71% with higher education) correctly identified the fallopian tube as the part of the genital tract where fertilisation usually takes place (25). These relatively good results regarding “technical” aspects of reproduction may result from study sampling that favoured populations with or during university education (and in the case of the present study—during medical or health-related university education). On the other hand, such results may suggest that Polish education focuses on the mechanistic model of the human body rather than on making RH education practical, close to students' experience, and delivered—as international standards (35) recommend—in an interactive way and with systematic youth participation. In practice, Polish children and adolescents are taught the basics of human reproduction in biology classes. In addition, there is a subject called education for family life (EFL) introduced in 1999 for pupils aged 9–10 and above until the completion of secondary education. Its curriculum includes sexual and RH education, but tends to concentrate on traditional family values and roles. Although it is obligatory for schools to provide 14 h of EFL a year, it is optional for pupils to attend the classes. The subject is often neglected by schools and disparaged by students. The attendance in primary schools reaches 73%, but only 37–51% in different kinds of secondary schools (36). The teaching methods reported by the attendees are basically lectures (90%), as well as film presentations (48%), discussions (44%), and team work (32%). However, only 87% of the attendees felt they were allowed to ask questions, and 40% were not permitted to discuss anything with either the teacher or classmates. Fifty-five percent felt they were allowed to express their opinions freely (37). Such a learning environment is hardly conducive to convincing young people that RH is relevant to them and constitutes a vital part of their lives. Selected elements of RH education (mostly natural family planning methods) are provided to would-be spouses at premarital family counselling meetings required by the Catholic Church before concluding a marriage. The median age of a Polish woman contracting the first marriage is nearly 28 years (38), which means that this additional education comes quite late for many young women. Therefore, it can be assumed that EFL classes often remain their primary source of RH information until they become university students.

The results of the present study also point to the differences in reliability and quality of the information obtained from various sources (see Additional File 2). As can be seen, almost a half of the study participants indicated using a few sources of information. This group achieved quite good results. Of the other half that indicated single sources, most relied on middle or high school classes, with mediocre results—an indication that formal school education is failing. The use of other sources, including university courses, was reported by 8.3% of the students and produced the best results. The students who relied on either the media or health professionals had relatively good knowledge as well. The participants who were informed by peers scored much poorer than those informed by parents, but it should be noted that both the groups were small and the conclusions should be treated with caution. In contrast, American females (34) claimed that they acquired RH knowledge from their gynaecologists (49%), then from family and friends (29%), from the Internet (17%), from their general practitioners (16%), and from other sources. Australian women (39) most often looked for information on the Internet and in books, while only 18% of Australians obtained it from doctors. The Internet is a very popular source of health-related information in Poland (27, 40–42) as well as abroad (39, 43–47), but the quality and reliability of the information presented there varies a lot. Since the use of only one source of information was not sufficient for our respondents, it seems crucial to ensure that the available sources provide quality and up-to-date information. It seems that even in the groups where motivation to expand knowledge is high, the level of knowledge may be insufficient owing to poor quality information. For example, while 86.8% of the interviewed patients of ART clinics actively tried to improve their FA using various sources of information and 68.2% attempted timed intercourse within the fertile window of the menstrual cycle, only 12.7% were able to identify this window correctly (39).

Finally, the results of this study may indirectly point to the gap between the participants' knowledge and their practices. Even though this study did not explore the participants' practices in the sphere of RH, some conclusions about them can be reached by comparing our results with practices of Polish women reported by other researchers. For instance, the present results show that Polish women are aware of age-related fertility decline or at least of the optimum age for a woman to become a first-time mother. In the present study, the optimum age was defined as the biological peak of female fertility with the shortest waiting time to pregnancy (48, 49). In another Polish study by Deluga and Wiśniewska, 85.8% of the interviewees knew correctly when the best time for having the first child was. Yet, only 29.7% of the respondents declared that they intended to give birth to their first child before 26 years of age (27). Demographic data from Poland confirm the tendency to either postpone or forgo parenthood. The mean age of first-time mothers rose in Poland from 23.7 to 27.2 between 1995 and 2016 (50), reflecting a similar trend in OECD countries, where the mean between the same years rose from 26.0 to 28.9 (51). The median age of Polish mothers at first birth in 2016 was 29.9 (50). At the same time, the interval between the births of the first and the second child in Poland rose from 3.5 years for women born in the years 1960–1964 to 4.7 years for the 1975–1979 cohort (15). Between 2010 and 2014, the proportion of Polish childless women planning to become mothers later than in the next 3 years increased from 44 to 52% (15). Unfortunately, a marked increase of waiting time to pregnancy can be observed in women aged over 35 years. In 2014, the waiting times of a year or more were observed in only 4–5% of first-time Polish mothers aged 25–29, but in as many as 25% of mothers aged 35+ (49). Since women's knowledge of the optimum childbearing age is not enough to change their decisions, it is imperative to educate them on the factors which may help to maintain their reproductive potential beyond the optimum age. The present study demonstrated the women's limited knowledge of the factors adversely affecting fertility, thus pointing to a big gap to fill in Polish RH education.

People's reluctance to treat RH as a personally relevant issue can sometimes be observed in global FA research. As a result of such an attitude, personal risks tend to be underestimated, while chances of success—overestimated. For instance, the fertility clinic patients who took part in the study by Homan and Norman (33) correctly identified obesity as an infertility risk factor, yet a half of the obese women in the sample did not find their weight to be a factor affecting their own fertility. Another interesting example given by Pedro et al. was an observation that high awareness of age-related fertility decline was frequently accompanied by a belief that the decline starts later than it actually does. In addition, the chances of achieving pregnancy both spontaneously and through fertility treatment were often overestimated (24). Canadian researchers (32) discovered that 90.3% of the interviewed childless women knew about the age-related fertility decline, but 72.9% believed that good health and fitness in women aged over 30 years is a better indicator of fertility than age. Ottawa students surveyed in another Canadian study (52) overestimated fertility of women in their thirties as well as success rates of assisted reproductive technologies. The overoptimistic perception of parenthood chances were also observed in the USA (34), Denmark (53), Sweden (54), Nigeria (55), and Australia (56).

Another difference between knowing and doing that follows from the comparison of the present results with the available research is the neglect of primary and secondary disease prevention among Polish women of reproductive age. While the vast majority (93.4%) of our respondents were aware of the adverse effect of diseases on fertility, only about a half of young Polish women report attending gynaecological check-ups on a regular basis, and the other half make an appointment only when they have a problem or urgently need a consultation (27). Contrary to the Polish clinical care guidelines recommending that the initial routine gynaecologic visit should take place between the ages of 12 and 15 years (57), only a small proportion of Polish women (16.4%) have it before the age of 16 (58). What is more, young Polish women are affected by a number of lifestyle-related risk factors for non-communicable diseases and infertility. Although the prevalence of tobacco smoking in women aged 20–29 has fallen from 21% in 1996 to 18.7% in 2018, the falling trend has slowed down in the last few years (59). Approximately one third of Polish women aged 20–35 are overweight or obese (60, 61). About 1 in 2 Polish students report not having enough sleep, largely due to poor sleep hygiene and bedtime procrastination, which is more prevalent in students than in non-students, and in women than in men (62, 63). It appears that the knowledge of young Poles does not always translate into practices.

Although the present study provided an interesting picture of young Polish women, the authors admit that its design had some limitations. The first was the use of the questionnaire specially developed for the purposes of this study instead of a standardised questionnaire, which makes the findings more difficult to compare with other research on FA and RH knowledge. The second limitation was the use of a convenient sample, which limited the generality of the study.

The findings presented here suggest that the overall RH knowledge of young Polish female students is limited and patchy. As can be expected, the best knowledge can be found in medical university students and in the oldest age group. There is strikingly poor awareness of some fertility-compromising behaviours such as unbalanced diet, excessive physical effort and irregular sleep. It may indicate that the young women do not realise how these lifestyle-related factors may influence their present and future lives. What is more, in the light of other research, it seems that the theoretical knowledge does not translate into practices even in the areas where awareness is relatively high. Education which is currently available may have limited effect on behaviours and decisions related to reproductive health and, consequently, on redressing fertility gap and population decline.

Tackling these problems requires using a number of diverse strategies tailored to address the needs of the Polish population. As it has been stated above, the available research indicates that there is a need in Poland for multi-faceted activities targeting primarily economic instability, work-family tensions, and health problems (including infertility), which seem to be the three main obstacles to childbearing in Poland. As regards the first two kinds of solutions, a few reforms have been implemented within the last 10 years: the extension of paid maternal leave (partly transferable to fathers) and parental leave to a total of 52 weeks, extension of institutional care for children under 3 years old, and introduction of a generous monthly child allowance for every child. Young Polish parents interviewed by Suwada (64) considered these solutions to be helpful, but insufficient and in need of integrating with better gender equality policies, labour market policies, and housing policies.

Regarding the third type of solutions, i.e., RH promotion and education, Polish policymakers have only recently realised its importance. The national public health policy paper called the National Health Programme for the years 2016–2020 formulates 6 main goals of the Polish public health to be achieved through intersectoral collaboration. One of the goals is “contributing to improved reproductive health” (65, 66). Two out of the five activities included in this goal are related to RH research and guideline development, and the remaining 3 activities are closely connected with evidence-based RH education of the general public as well as would-be and current health professionals and educators. Unfortunately, only 1.03% of all the activities undertaken as part of the Programme in the years 2016–2018 were dedicated to the RH goal (67). It may suggest that the need for action has been recognised in Poland, but the urgency of the action probably has not.

High-quality health education is necessary for turning mere health knowledge into health literacy defined as the ability not only to read, understand, and apply new information, but also to “exert greater control over life events and situations” (68). High-quality, equitable (69), and widely available health education is necessary for making informed choices. Interventions aimed at increasing health literacy and tailored to patients' needs have been found to be effective or at least promising tools for changing health knowledge and behaviours (70–72). That is why it is imperative to further explore the gaps in RH education in Poland in order to make it more operational and practical, more interesting, and relevant to young people's everyday experience, and more comprehensive in terms of balancing the present focus on family values and pregnancy prevention with the content aimed at improving their FA and teaching them to look after their reproductive potential. Since nearly a third of the study participants relied on the information obtained during secondary school classes, it is advisable to pay special attention to examining and subsequent redesigning of the curricula of these classes. To redress the knowledge gaps observed in current university students, it should be ensured that medical university courses provide thorough RH/FA knowledge. It is particularly important with respect to would-be gynaecologists, who might become a more trusted source of evidence-based information for their patients if they were better trained in terms of health education skills and RH literacy. At other universities, elective courses should be arranged to advocate health-promoting and health-protective behaviours and to encourage young people to broaden their knowledge with the help of reliable sources of information such as health professionals. Relevant graduate, postgraduate and in-service courses should be available to future and present professionals responsible for spreading RH knowledge: teachers, health educators, school counsellors, and psychologists. The key messages of RH education should be the fact that our RH is a function of our general health status, and that our lifestyles directly influence them both.

## Plain English Summary

Poland is experiencing a decline in fertility: women tend to have fewer children and postpone motherhood to their 30s and 40s, which may cause problems getting pregnant. That is why especially young people should have knowledge sufficient to enable them to take care of their reproductive health. We decided to assess their knowledge by surveying Polish female university students. Four hundred and fifty-six students completed a questionnaire testing the knowledge of female and male reproductive physiology. In general, the students' knowledge was found to be incomplete. Better results were observed in the oldest age group and among medical university students. Over 90% of the respondents knew some fertility-compromising risks (smoking, diseases, stress), but few were aware of the adverse effect of unbalanced diet, irregular sleep, and long-lasting physical effort. Nearly a third said their only source of reproductive health knowledge was primary or secondary school classes. Therefore, it is crucial to provide high quality education at this level. Also university students as well as present and future teachers and health educators should be offered additional reproductive health courses. The education in this area should be as practical as possible to convince young people of the importance of looking after one's reproductive health.

## Data Availability Statement

The raw data supporting the conclusions of this article will be made available by the authors, without undue reservation.

## Ethics Statement

Ethical review and approval was not required for the study on human participants in accordance with the local legislation and institutional requirements. The patients/participants provided their written informed consent to participate in this study.

## Author Contributions

EC, BK, and AL conceived its idea. BK and RS worked on the statistics and tables. BK and JK wrote the core of the manuscript. EC coordinated its further development. EC and AL contributed to the discussion and developed the figure. All authors made substantial contributions to the conception and design of this paper, read, revised, and approved the final manuscript.

## Conflict of Interest

The authors declare that the research was conducted in the absence of any commercial or financial relationships that could be construed as a potential conflict of interest.

## Abbreviations

ANOVA: analysis of variance
ART: assisted reproductive technology
EFL: education for family life
FA: fertility awareness
Q: question (in the survey questionnaire)
RH: reproductive health
STI: sexually transmitted infection
TFR: total fertility rate.
