# Supplementary material for: Reproductive Health Literacy and Fertility Awareness Among Polish Female Students
Source: Front Public Health. 2020 Sep 11;8:499. doi: 10.3389/fpubh.2020.00499 (PMC7516014; doi:10.3389/fpubh.2020.00499)
Supplement: Supplementary file 1 [file Data_Sheet_1.DOCX]

**Questionnaire**

1. What is fertility? ……………………………………………………………………………………………………
2. How long does a normal menstrual cycle last?
3. always exactly 28 days
4. 21-35 days
5. 26-28 days
6. none of the above answers is correct
7. I don’t know
8. Which day counts as the first day of the menstrual cycle?
9. the first day of bleeding
10. the day of ovulation
11. the last day of bleeding
12. I don’t know
13. What is ovulation?
14. another name for a menstrual cycle
15. menstrual bleeding
16. a release of an ovum from an ovarian follicle
17. I don’t know
18. Does ovulation occur in each cycle?
19. not necessarily
20. yes, always
21. it occurs in regular cycles but not in irregular ones
22. I don’t know
23. When does ovulation occur during the menstrual cycle?
24. around 14 days before menstrual bleeding
25. always on the 14^th^ day of the cycle
26. exactly in the middle of the cycle, irrespective of its length
27. I don’t know
28. Does the body temperature change during the menstrual cycle depending on its phase?
29. it may (but need not) change
30. yes, it always changes
31. I don’t know
32. How long does an ovum live?
33. from ovulation to the end of the cycle
34. from the first day of bleeding to the 14^th^ day of the cycle
35. 12 – 24 hours
36. 96 hours
37. I don’t know
38. Which phase of the menstrual cycle is the most fertile?
39. the luteal phase
40. the ovulatory phase
41. the follicular phase
42. I don’t know
43. A sexual intercourse is the most likely to lead to pregnancy when (you can choose one or more answers):
44. libido increases
45. cervical mucus is whitish and sticky
46. the cervix is soft
47. menstrual pain occurs
48. cervical mucus is clear and stretchy
49. ovulation pain occurs
50. the cervix is hard
51. When is a woman fertile during her lifetime?
52. 15-35 years of age
53. from the first to the last menstruation
54. from the first to the last ovulation
55. I don’t know
56. What is the best age for a woman to have the first baby?
57. under 18 years of age
58. over 35 years of age
59. 20-25 years of age
60. over 25 years of age
61. Is a woman with irregular cycles infertile?
62. yes, always
63. no, fertility is not conditional on regular cycles, or vice versa
64. not always – having regular cycles is not the only indicator of fertility
65. I don’t know
66. What is menopause?
67. the last menstruation followed by 12 consecutive months without menstruation
68. hot flashes and sweating
69. the period in a woman’s life (a few months or years long), when her fertility gradually ceases
70. the period in a woman’s life from the last menstruation to death
71. I don’t know
72. Which factors may have an ADVERSE effect on human fertility? (you can choose one or more answers)
73. smoking
74. irregular circadian rhythms
75. overeating
76. diseases
77. eating a lot of vegetables (4 times a day)
78. stress
79. drastic diet changes
80. long-lasting physical effort
81. non-professional sports activity
82. frequent sexual intercourses
83. full-time work
84. How long does a healthy sperm live in favourable conditions?
85. 24 hours
86. 7-8 days
87. 3-5 days on average
88. I don’t know
89. How long is a healthy man fertile in his lifetime?
90. his whole life
91. between 15 and 55 years of age
92. from the first ejaculation to about 75 years of age
93. from reaching full maturity to death
94. I don’t know
95. Do you have any children? ……………………………
96. If yes, were your pregnancies planned? ……………………………
97. What are your sources of information on fertility? (you can choose one or more answers)
98. healthcare professionals
99. middle and secondary school classes
100. peers
101. parents
102. media
103. other sources, which? ……………………………

DEMOGRAPHIC INFORMATION

1. University
2. University department
3. Course of study
4. Year of study
5. Age
6. Place of residence
