# Supplementary material for: Reproductive Health Literacy and Fertility Awareness Among Polish Female Students
Source: Front Public Health. 2020 Sep 11;8:499. doi: 10.3389/fpubh.2020.00499 (PMC7516014; doi:10.3389/fpubh.2020.00499)
Supplement: Supplementary file 2 [file Data_Sheet_2.docx]

| **ADDITIONAL FILE 2 \|** Sources of information vs. students’ knowledge. | | | | | | | | | | | | | |
| --- | --- | --- | --- | --- | --- | --- | --- | --- | --- | --- | --- | --- | --- |
|  | | **Sources of information (percentage of participants who indicated the source)** | | | | | | | | | | | |
|  | | **Mean** | | **Health professionals** | | **Middle or high school classes** | **Peers** | **Parents** | **Media** | **Other sources** | **A number of sources** | **No source indicated** |  |
| **Knowledge* of:** | | **n=456** | | **(5.0%)** | | **(28.3%)** | **(1.5%)** | **(1.8%)** | **(5.3%)** | **(8.3%)** | **(47.1%)** | **(2.6%)** | ***p***** |
| Q4. definition of ovulation | | 97.8% | | 100.0% | | 98.4% | 71.4% | 100.0% | 95.8% | 97.4% | 98.1% | 100.0% | ≤0.001 |
| Q6. timing of ovulation | | 59.2% | | 52.2% | | 50.4% | 42.9% | 25.0% | 79.2% | 73.7% | 62.8% | 50.0% | ≤0.05 |
| Q7. cycle temperature changes | | 40.4% | | 39.1% | | 38.8% | 28.6% | 87.5% | 45.8% | 31.6% | 41.4% | 33.3% | 0.0445 |
| Q11. length of female lifetime fertility | | 58.8% | | 56.5% | | 54.3% | 28.6% | 62.5% | 54.2% | 71.7% | 65.1% | 25.0% | ≤0.05 |
| Q12. best time for first child | | 93.4% | | 87.0% | | 92.2% | 71.4% | 100.0% | 87.5% | 94.8% | 95.8% | 91.7% | 0.012 |
| Q14. definition of menopause | | 20.8% | | 47.8% | | 13.2% | 28.6% | 0.0% | 12.5% | 50.0% | 18.6% | 25.0% | ≤0.001 |
| Q15b. adverse factors: irregular circadian rhythms (T) | | 60.3% | | 65.2% | | 48.8% | 42.9% | 62.5% | 70.8% | 81.6% | 62.3% | 58.3% | ≤0.05 |
| Q15h. adverse factors: long-lasting physical effort (T) | | 37.50% | | 56.5% | | 27.9% | 57.1% | 37.5% | 20.8% | 50.0% | 40.9% | 25.0% | ≤0.05 |
| Q15k. adverse factors: full-time work (F) | | 92.30% | | 73.9% | | 94.6% | 71.4% | 87.5% | 95.8% | 86.8% | 94.9% | 83.3% | ≤0.05 |
| **As a proportion of correct answers.* | | | | | | | | | | | | | |
| *** Only the statistically significant results are presented.* | | | | | | | | | | | | | |
| *T, true; F, false.* |  | |  | |  | |  |  |  |  |  |  |  |
