# Supplementary material for: Reproductive Health Literacy and Fertility Awareness Among Polish Female Students
Source: Front Public Health. 2020 Sep 11;8:499. doi: 10.3389/fpubh.2020.00499 (PMC7516014; doi:10.3389/fpubh.2020.00499)
Supplement: Supplementary file 3 [file Data_Sheet_3.docx]

| **ADDITIONAL FILE 3 \|** Knowledge differences between students of different universities. | | | | | | | | |
| --- | --- | --- | --- | --- | --- | --- | --- | --- |
|  | **University (percentage of participants from a given university)** | | | | | | | |
|  | **Mean** | **PUT** | **PULS** | **AMU** | **PUEB** | **AMP** | **PUMS** | ***p***** |
| **Knowledge* of:** | **n=456** | **(12.1%)** | **(12.7%)** | **(16.7%)** | **(12.7%)** | **(6.8%)** | **(39%)** |  |
| Q3. first day of cycle | 86.0% | 81.8% | 79.3% | 84.2% | 79.3% | 71.0% | 94.9% | ≤0.001 |
| Q4. definition of ovulation | 97.1% | 96.4% | 100.0% | 93.4% | 100.0% | 93.5% | 99.4% | 0.0089 |
| Q5. frequency of ovulation | 48.0% | 56.4% | 50.0% | 36.8% | 43.1% | 29.0% | 71.9% | ≤0.001 |
| Q9. most fertile cycle phase | 70.9% | 69.1% | 72.5% | 67.1% | 63.8% | 71.0% | 82.0% | 0.001 |
| Q10b. fertility signs: whitish and sticky mucus (F) | 73.9% | 67.3% | 56.9% | 72.4% | 69.0% | 58.1% | 87.1% | ≤0.001 |
| Q10c. fertility signs: soft cervix (T) | 46.9% | 34.5% | 46.6% | 43.4% | 44.8% | 25.8% | 56.2% | ≤0.05 |
| Q10e. fertility signs: clear and stretchy mucus (T) | 59.4% | 50.9% | 53.4% | 50.0% | 43.1% | 38.7% | 76.4% | ≤0.001 |
| Q10g. fertility signs: hard cervix (F) | 91.2% | 81.8% | 91.4% | 89.7% | 96.6% | 77.4% | 94.9% | ≤0.001 |
| Q11. length of female lifetime fertility | 57.5% | 52.7% | 70.7% | 44.8% | 62.1% | 51.6% | 62.9% | 0.0184 |
| Q14. definition of menopause | 11.5% | 1.8% | 3.4% | 5.3% | 5.2% | 6.5% | 46.6% | ≤0.001 |
| **As a proportion of correct answers.* | | | | | | | | |
| *** Only the statistically significant results are presented.*  *T, true; F, false; PUT - Poznan University of Technology; PULS- Poznan University of Life Sciences; AMU - Adam Mickiewicz University; PUEB - Poznan University of Economics and Business; AMP - Academy of Music in Poznan; PUMS - Poznan University of Medical Sciences* | | | | | | | | |
|  | | | | | | | | |
